# High Sensitivity Refractive Index Sensor Based on Dual-Core Photonic Crystal Fiber with Hexagonal Lattice

**DOI:** 10.3390/s16101655

**Published:** 2016-10-08

**Authors:** Haiyang Wang, Xin Yan, Shuguang Li, Guowen An, Xuenan Zhang

**Affiliations:** College of Information Science and Engineering, Northeastern University, Shenyang 110819, China; wanghaiyangneu@gmail.com (H.W.); lishuguang@ise.neu.edu.cn (S.L.); guowen_an@yeah.net (G.A.); zhangxuenan@ise.neu.edu.cn (X.Z.)

**Keywords:** photonic crystal fiber, refractive index sensor, high sensitivity

## Abstract

A refractive index sensor based on dual-core photonic crystal fiber (PCF) with hexagonal lattice is proposed. The effects of geometrical parameters of the PCF on performances of the sensor are investigated by using the finite element method (FEM). Two fiber cores are separated by two air holes filled with the analyte whose refractive index is in the range of 1.33–1.41. Numerical simulation results show that the highest sensitivity can be up to 22,983 nm/RIU(refractive index unit) when the analyte refractive index is 1.41. The lowest sensitivity can reach to 21,679 nm/RIU when the analyte refractive index is 1.33. The sensor we proposed has significant advantages in the field of biomolecule detection as it provides a wide-range of detection with high sensitivity.

## 1. Introduction

Photonic crystal fiber, which is also called micro-structured optical fiber, has many advantages such as endless single mode, ultra low loss, high birefringence and high nonlinearity [1,2,3,4]. Due to its particular optical properties compared with conventional fibers, PCF has been applied in many optical devices such as fiber lasers, optical communications and fiber sensors [5,6,7]. The refractive index sensor based on PCF has attracted considerable attention to the potential in the remote real-time detection recently. With the improvement of fabrication technology and sensing technique on detecting the refractive index, PCF sensors have widely used in many aspects of the chemical and biological detection industry [8,9,10].

Most of PCF sensors are based on surface plasmon resonance (SPR), photonic bandgap properties of the PCF or resonant coupling. In 2009, Shi provided a refractive index sensor based on photonic bandgap fiber with long period grating and demonstrated a sensitivity of 17,900 nm/RIU [11]. In 2012, Tian proposed PCF-based SPR sensors and its numerical results showed that the refractive index sensitivity of the sensor is 7300 nm/RIU [12]. In 2013, Xiao investigated a refractive index sensor based on the resonant coupling mechanism and its numerical results revealed that the sensitivity of the sensor could reach to 17,250 nm/RIU [13].

From the sensors mentioned above, only one-core PCF was used. However, due to the design flexibility and the matured stack-and-draw fabrication technology, multi-core PCFs including dual-core PCFs have showed excellent performances in special applications such as polarization splitter, polarization filter and refractive index sensors [14,15,16]. In 2012, Shuai numerically characterized a multi-core PCF-based sensor which average sensitivity is 2929.39 nm/RIU in the sensing range of 1.33–1.42 [17]. We know that the refractive index detection can be implemented by resonant coupling in dual-core PCF. The detection sensitivity of the sensor can be effectively improved by filling the analytes into the central air holes [18]. Moreover, both wide-range detection and high sensitivity can be achieved by using the exponential dependence of inter-core coupling on analyte refractive index [19].

Refractive index sensors have attracted a lot of attention in the field of biomolecule detection as they provide a wide-range of detection with high sensitivity. Rindorf demonstrated the long-period gratings in PCF can be used for biological sesnsing by filling the biological molecule into the air holes and measured the thicknesses of a monolayer of poly-L-lysine and double-stranded DNA [20]. Jensen demonstrated selective detection of *α*-streptavidin or *α*-CRP antibodies in microstructured polymer optical fibers by a sensor layer of complementary biomolecules immobilized inside the air holes [21].

In this paper, we design a refractive index sensor based on dual-core photonic crystal fiber with a hexagonal air-hole lattice structure. For the dual-core PCF sensor we proposed, the sensitivity is calculated according to the energy coupling between two cores. The sensitivity of the sensor we designed is much higher. The sensitivity of the sensor we proposed can reach to 21,679–22,983 nm/RIU with a dynamic refractive index ranging of 1.33–1.41. Besides, we only filled analytes into the air holes to detect the refractive index of the analytes. It is much easier to manufacture, which is a better candidate for refractive index sensor devices.

## 2. The Structure and Theoretical Analysis

**Figure 1:**
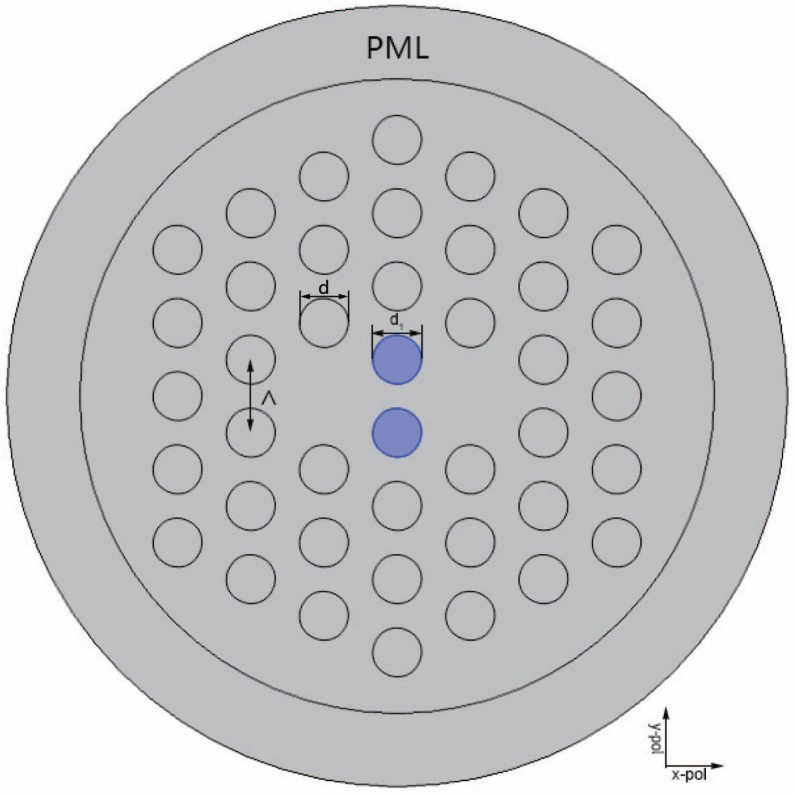
Cross-section of the proposed refractive index sensor.

The cross section of the proposed sensor with hexagonal air-hole lattice is shown in Figure 1. The properties of the proposed sensor have been analyzed with the FEM by using the COMSOL Multiphysics software [22]. A perfectly matched layer (PML) and a scattering boundary condition are used to decrease the energy loss [23]. The analyte is filled into two air holes located in the center to increase the area of the analyte which can greatly improve the sensitivity of the sensor. The refractive index of anlyate is $n_{a}$.

All the diameters of the air holes are *d* = *d*${}_{1}$ = 2 μm and the pitch of the adjacent air holes is Λ = 3 μm, as shown in Figure 1. The impact on the properties of the proposed sensor by changing the $d_{1}$ and Λ will be discussed later.

The background material is fused silica whose material dispersion can be obtained from the Sellmeier formula [24]:

(1) $$n^{2}\left(\omega \right)=1+\sum_{j=1}^{3}\frac{B_{j}\omega_{j}^{2}}{\omega_{j}^{2}-\omega^{2}}$$

where $B_{1}=0.6961663$, $B_{2}=0.4079426$, $B_{3}=0.8974794$, $\lambda_{1}=0.0684043\;\mu$m, $\lambda_{2}=0.1162414\;\mu$m, $\lambda_{3}=9.896161\;\mu$m, $\lambda_{j}=2\pi c/\omega_{j}$ and *c* is the speed of light in vacuum.

Regarding to the fabrication, the refractive index sensor we proposed is easier to manufacture compared with the complex structures mentioned in the previous literatures [25,26,27]. With the development of infiltration technique, the samples filled into the PCF have been illustrated both experimentally and theoretically [28,29]. It is possible to fabricate the proposed refractive index sensor in the practical production.

**Figure 2:**
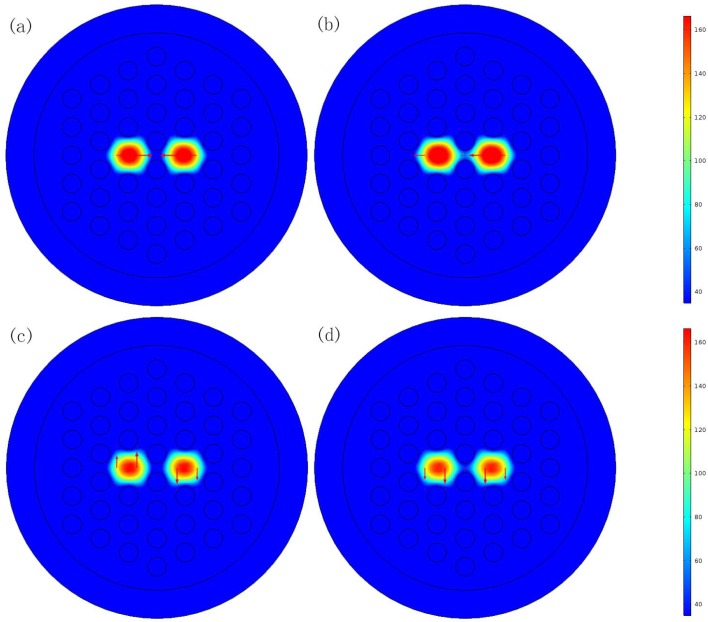
Electric filed distributions of the four supermodes in the PCF sensor: (**a**) the *x*-polarized odd mode (**b**) the *x*-polarized even mode (**c**) the *y*-polarized odd mode (**d**) the *y*-polarized even mode.

The two fiber cores of the PCF are formed by eliminating two central air holes in the horizontal direction. The anylate is filled into two central air holes in the vertical direction for detection. According to the coupling theory, the dual-core PCF has four supermodes in the *x*-polarization (*x*-even and *x*-odd) and the *y*-polarization (*y*-even and *y*-odd). Figure 2 shows the electric field distributions of the proposed sensor.

The coupling length of the dual-core PCF indicates the periodic variation of energy between the two cores, which can be defined by the formula [30]:

(2) $$L_{i}=\frac{\pi }{\beta_{e}^{i}-\beta_{o}^{i}}=\frac{\lambda }{2(n_{e}^{i}-n_{o}^{i})},\;i=x,y$$

where $\beta_{e}^{i}$ and $\beta_{o}^{i}$ are the propagation constants of i-polarized even and odd super modes, $n_{e}^{i}$ and $n_{o}^{i}$ are the effective refractive indexes of i-polarized even and odd super modes respectively.

**Figure 3:**
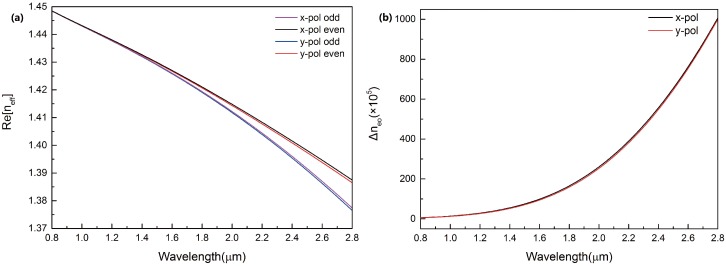
(**a**) The four lines are the effective refractive index of four supermodes of the dual-core PCF when $n_{a}=1.33$, $d_{1}=2\;\mu$m and Λ=3μm; (**b**) Black and red lines are the calculated effective refractive difference of the four supermodes when $n_{a}=1.33$, $d_{1}=2\;\mu$m and Λ=3μm.

The effective refractive index of the dual-core PCF was simulated, as shown in Figure 3a. With the increasing of wavelength, the effective refractive index of four supermodes decreases. The effective refractive index in *y*-polarization decrease faster than that in *x*-polarization. The variations of $\Delta n_{eo}=\left|n_{e}-n_{o}\right|$ with wavelength in *x*-polarization and *y*-polarization were simulated, as shown in Figure 3b. With the increasing of wavelength, the index difference ($\Delta n_{eo}$) increases and the index difference in *x*-polarization is larger than that in *y*-polarization.

**Figure 4:**
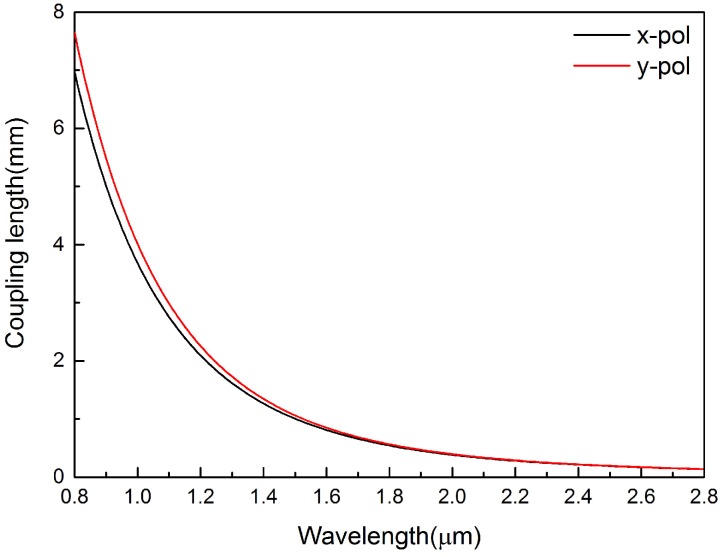
Black and red lines are the calculated coupling length of the dual-core PCF in *x*-polarization and *y*-polarization respectively when $n_{a}=1.33$, $d_{1}=2\;\mu$m and Λ=3μm.

Figure 4 shows the variation of coupling length with wavelength in *x*-polarization and *y*-polarization when the analyte refractive index is $n_{a}=1.33$. It is obvious that the coupling length decreases with the increasing of wavelength in *x*-polarization and *y*-polarization. Moreover, the coupling length in *y*-polarization is larger than that in *x*-polarization.

## 3. Results and Discussion

### 3.1. The Determine of the Transmission Length for the Refractive Index Sensor

**Figure 5:**
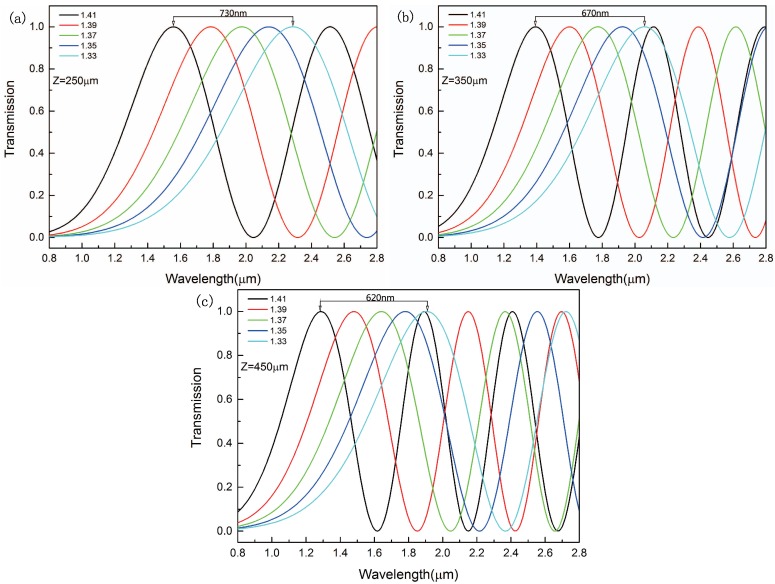
Transmission curve for the refractive index of the analyte in the range of 1.33–1.41 when the transmission length is (**a**) Z=250μm; (**b**) Z=350μm; (**c**) Z=350μm.

The transmission curves for *x*-polarized light with different transmission lengths were simulated, as shown in Figure 5. All of the wavelength peaks experiences a blue shift when the refractive index increases from 1.33 to 1.41. As we can see from Figure 5, when the transmission length is 250μm, 350μm and 450μm, the distance between the peaks is 730 nm, 670 nm and 620 nm respectively. With the increasing of transmission length from 250μm to 450μm, the distance between the peaks slightly decreases. It represents that the sensitivity of the sensor slightly decreases when the transmission length increases from 250μm to 450μm. We can also see from Figure 5c that the second peak in $n_{a}=1.41$ intersects with the peak in $n_{a}=1.33$ when the fiber length is 450μm. It is difficult to detect the refractive index when two peaks intersect in different refractive indices. In order to separate the two peaks to eliminate the influence, we choose that the transmission length is 250μm which can avoid intersection.

### 3.2. The Effect of the Adjacent Air Holes Pitches on Sensitivity

**Figure 6:**
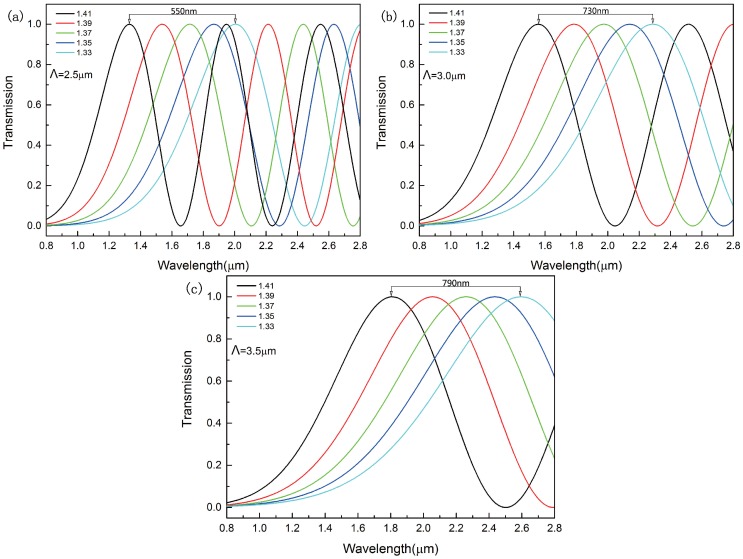
Transmission curve for the refractive index of the analyte in the range of 1.33–1.41 when the pitch of the adjacent air holes is (**a**) Λ=2.5μm; (**b**) Λ=3.0μm; (**c**) Λ=3.5μm.

In this section, we discuss the impact of the pitch of the adjacent air holes on sensitivity. The transmission length of the sensor is 250μm. Figure 6 shows the transmission curves in *x*-polarization when the pitch of the adjacent air holes changes from Λ=2.5μm to Λ=3.5μm. The wavelength peaks experience a blue shift when the refractive index of the analyte ranges from 1.33 to 1.41. With the increasing of the Λ, both the distance between the peaks and the period of transmission curves increase. The distance between the peaks increases from 550 nm to 730 nm when the pitch of the adjacent air holes changes from Λ=2.5μm to Λ=3.0μm. However, the distance increases only from 730 nm to 790 nm when Λ changes from 2.5μm to 3.0μm. The distance between the peaks is crucial to the sensitivity. With the increasing of the distance between the peaks, the sensitivity can be higher. However, the distance between the peaks increases slowly. The proposed sensor detects the refractive index mainly according to the wavelength peaks. Obviously, the shorter period is more beneficial to wavelength measurement and vice versa. It is not the best way to improve the sensitivity by increasing the pitch of the adjacent air holes.

### 3.3. The Effect of the Size of the Analyte-Filled Air Holes on Sensitivity

**Figure 7:**
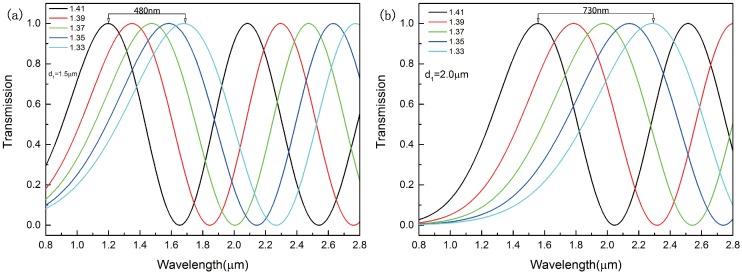

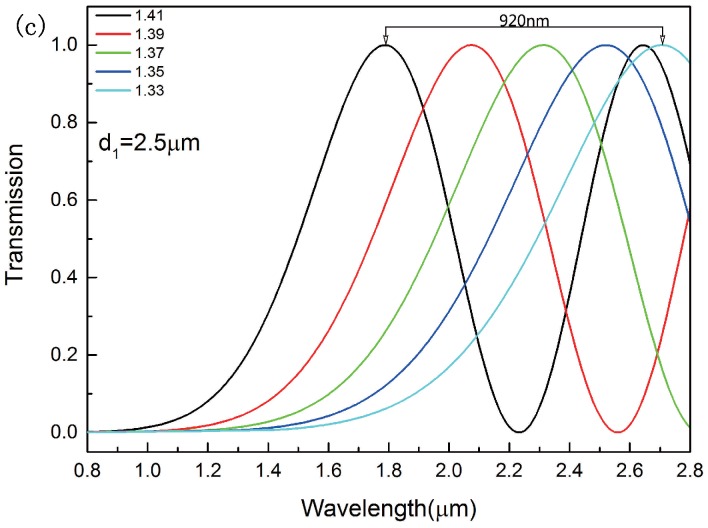
Transmission curve for the refractive index of the analyte in the range of 1.33–1.41 when the diameter of analyte-filled air holes is (**a**) $d_{1}=1.5\;\mu$m; (**b**) $d_{1}=2.0\;\mu$m; (**c**) $d_{1}=2.5\;\mu$m.

The diameter of the analyte-filled air holes $d_{1}$ is a key parameter for the sensitivity of the proposed sensor. Figure 7 shows the transmission curves in *x*-polarization with different diameters of analyte-filled air holes. The transmission length of the sensor is also 250μm. All the wavelength peaks experience a red shift when the diameter of $d_{1}$ changes from 1.5μm to 2.5μm. With the increasing of $d_{1}$, the distance between the peaks increases significantly. When the diameter of $d_{1}$ changes from 2.0μm to 2.5μm, the distance between the peaks increases from 730 nm to 920 nm. When the diameter of $d_{1}$ changes from 1.5μm to 2.0μm, the distance between the peaks increases form 480 nm to 730 nm. It means that the sensitivity of the sensor increases markedly. Furthermore, the refractive index of the analyte can be measured more accurately due to the constant period. It is the most effective way to improve the sensitivity by increasing the diameter of the analyte-filled air holes.

### 3.4. The Result of Numerical Fitting and Sensitivity Calculation

We calculate the sensitivity by using the shift of the peaks with the variation of the analyte refractive index. The refractive index sensitivity S can be written as:

(3) $$S_{\lambda }\left(nmRIU^{-1}\right)=\Delta \lambda_{peak}/\Delta n_{a}$$

where $\lambda_{peak}$ is the shift of the transmission curve and $\Delta n_{a}$ is the variation of the analyte refractive index.

**Figure 8:**
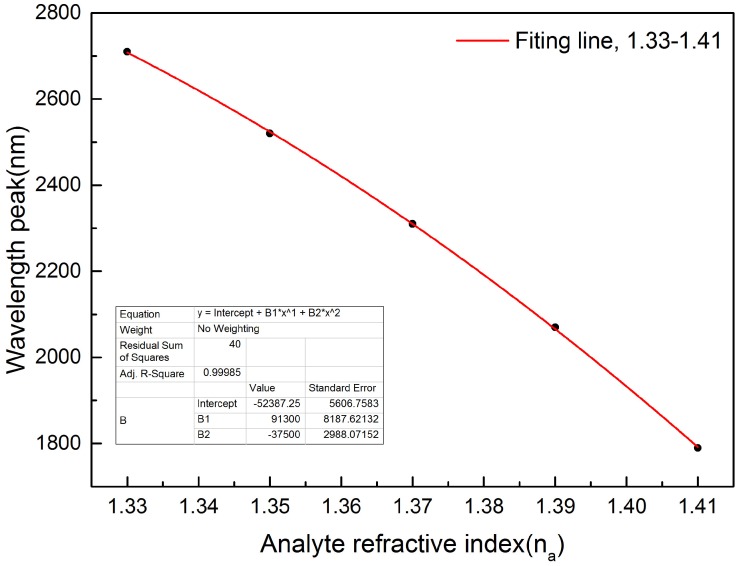
Numerical fitting result of function between analyte refractive index and wavelength peak when $d_{1}=2.5\;\mu$m and other parameters are fixed.

Figure 8 shows the numerical fitting result. The slope of the curve stands for the sensitivity of the proposed sensor. The fitting equation and value are shown in the inset of Figure 8. Calculation result shows that the highest sensitivity of *x*-polarized light is 22,983 nm/RIU when the analyte refractive index is $n_{a}=1.41$ at the operate wavelength of 1.79μm. The lowest sensitivity of *x*-polarized light is 21,679 nm/RIU when the analyte refractive index is $n_{a}=1.33$ at the operate wavelength of 2.71μm. It is much higher than the highest sensitivity among the refractive index sensors mentioned in the previous literatures [31,32,33]. The sensor we proposed have a good prospect on sensor devices due to the high sensitivity. Moreover, we calculate the highest sensitivity of *y*-polarized light is 20,014 nm/RIU with the analyte refractive index is $n_{a}=1.41$. The sensitivity in *x*-polarized light is larger than that in *y*-polarized light, so we adopt *x*-polarized light as the analyze mode.

If the instrumental peak-wavelength resolution is assumed to be $\Delta \lambda_{min}=0.1$ nm, the refractive index resolution of the corresponding sensor can be obtained as:

(4) $$R=\Delta n_{a}\Delta \lambda_{min}/\Delta \lambda_{peak}$$

where $\Delta n_{a}$ = 0.02, $\Delta \lambda_{peak}$ = 190 nm, 210 nm, 240 nm and 280 nm respectively when refractive index changes from 1.33 to 1.41. According to the parameters mentioned above, the refractive index resolution we calculate is 1.05 ×$10^{-5}$ RIU, 9.52 ×$10^{-6}$ RIU, 8.33 ×$10^{-6}$ RIU and 7.14 × $10^{-6}$ RIU respectively.

The refractive index sensor we proposed can achieve quantitative detection by detecting small change in the analyte refractive index. We can detect the change of the information of the biological molecule reaction by measuring the wavelength. The sensor will have broad application in many fields such as pathogens, toxins, drug residues, vitamins, antibodies, proteins and parasites as it can provide high sensitivity, label-free and wide-range detection.

## 4. Conclusions

A refractive index sensor based on dual-core photonic crystal fiber with hexagonal lattice has been proposed. Numerical analysis of the proposed structure is carried out with FEM. The properties of the refractive index sensor are discussed and numerical results show that the optimal sensitivity of the structure can be up to 22,983 nm/RIU when the refractive index of the analyte is $n_{a}=1.41$. The lowest sensitivity can reach to 21,679 nm/RIU when the refractive index of the analyte is $n_{a}=1.33$. Moreover, good resolution of $10^{-6}$ RIU is achieved for the proposed structure. Both wide-range and high sensitivity making it possible to achieve real-time, fast and convenient detection.
